# Alveolar Crestal Approach for Maxillary Sinus Membrane Elevation with <4 mm of Residual Bone Height: A Case Report

**DOI:** 10.1155/2018/1063459

**Published:** 2018-06-28

**Authors:** Jae Won Jang, Hee-Yung Chang, Sung-Hee Pi, Yoon-Sang Kim, Hyung-Keun You

**Affiliations:** Department of Periodontology, School of Dentistry, Wonkwang University, 344-2 Shinyong-dong, Iksan, Jeonbuk 54538, Republic of Korea

## Abstract

**Introduction:**

For maxillary sinus membrane elevation (MSME), the lateral window approach and crestal approach are available, and high success rates have been achieved with low residual bone height as a development of technology.

**Objective:**

To evaluate MSME using the crestal approach with a rotary-grind bur (RGB (including reamer or sinus bur)) in patients with residual bone height of <4 mm.

**Materials and Methods:**

Ten implants were placed in 10 patients with residual bone height of <4 mm, by sinus elevation using an RGB. The implant stability quotient (ISQ) was measured immediately after implant placement (ISQ 1) and before taking impression for the final prosthesis (ISQ 2). The extent of marginal bone loss was measured on periapical radiographs.

**Results:**

The mean residual bone height before implant placement was 3.41 ± 0.53 mm; no complications, including membrane perforation, severe postoperative pain, or discomfort, occurred either during or after surgery. The mean ISQ 1 was 63.4 ± 12.1, whereas the mean ISQ 2 was 77.6 ± 5.8. The mean marginal bone resorption was 0.23 ± 0.18 mm on periapical radiographs.

**Conclusions:**

MSME using the crestal approach with an RGB is a reliable technique for implant placement in sites where available bone is insufficient.

## 1. Introduction

A reduction in alveolar bone, through sinus pneumatization in the maxillary posterior area, is commonly encountered after tooth extraction. Maxillary sinus membrane elevation (MSME) is an essential procedure to recover the appropriate bone height for implant treatment and has become a generalized clinical technique used by many dentists in recent years.

For MSME, the lateral window approach and the alveolar crestal approach through the extraction socket are both available; operations using either of these techniques have yielded high implant success rates [1, 2]. In previous studies, the lateral window approach has been reported to elevate the maxillary sinus by up to 10−12 mm, which is greater elevation than that provided by the alveolar crestal approach (2.5–5.7 mm); notably, the lateral window approach is generally used in cases with low residual bone height (≤4-5 mm) [3–6]. However, the lateral window approach is technically more difficult than the alveolar crestal approach and is more likely to cause postoperative complications, including pain and swelling [7]. Moreover, it has a sinus membrane perforation rate of 12–40%, which is higher than that of the alveolar crestal approach (2.2–6.7%) [4–6, 8–12].

Since the alveolar crestal approach was introduced, several studies have reported high survival rates following the use of this technique [4, 13–16]. The osteotome technique, which avoids membrane perforation, has led to significant advances in implant treatment; however, there are disadvantages associated with the osteotome technique [3, 5], the most significant of which is that the patient may experience headaches and postoperative dizziness that result from aggressive mallet tapping [17].

To overcome these problems, instruments designed to grind the bone without perforating the membrane—rather than fracturing the maxillary cortical bone via mallet tapping—have been developed [6, 17, 18]. Compared with traditional methods, this method reportedly confers advantages to both the operator and the patient, as it is simple to perform and is associated with fewer postoperative complications [17, 19, 20]. This method of smoothly grinding the bone may enable the membrane to be elevated in a stable manner, even in areas with short residual bone height.

The objective of the present study was to assess the postoperative outcomes following MSME via the alveolar crestal approach, using a rotary-grind bur (RGB (including reamer or sinus bur)) in the maxillary posterior area of patients with residual bone heights <4 mm.

## 2. Materials and Methods

### 2.1. Participants

The present study included individuals who underwent implant placement with MSME via the alveolar crestal approach using an RGB; these individuals were recruited from among a group of patients who visited the Department of Periodontology at Wonkwang University Dental Hospital (Jeonbuk, South Korea) and who had residual bone height <4 mm in the maxillary posterior area. Patients with anatomical structures that would interfere with the use of alveolar crestal approach, such as sinus septum, were excluded from this study. The study was approved by the Institutional Review Board (IRB) of Wonkwang University Dental Hospital (WKDIRB 201702-01). A total of 10 patients (three males and seven females) participated in the study, and a total of 10 implants were placed using the alveolar crestal approach. The age of the participants ranged between 38 and 71 years (mean, 54.2 years) (Table 1). Although two participants were smokers, they were instructed to abstain from smoking for 2 weeks before and 2 months after the procedure.

**Figure 1:**
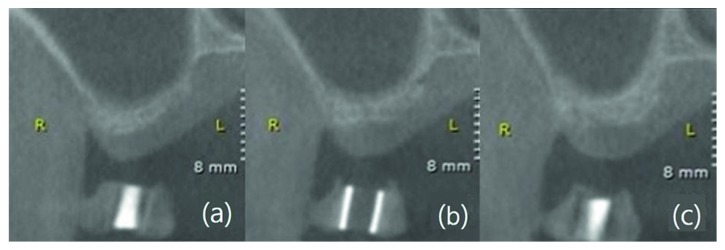
Residual bone height measurement method. Average value derived from (a) mesial, (b) central, and (c) distal aspects of the stent on computed tomography coronal view.

Residual bone height was measured as the distance from the alveolar crest to the sinus floor on the coronal view of a cone beam computed tomography (CT) image; it was expressed as the average value derived from the mesial, central, and distal aspects of the stent on the CT image (Figure 1).

### 2.2. Surgical Method

**Figure 2:**
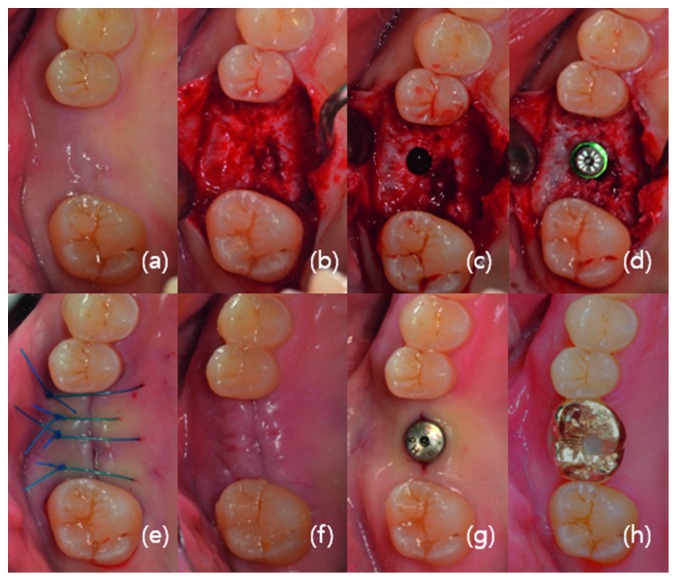
Implant treatment procedure. (a) Preoperative image; (b) immediately after full-thickness flap elevation; (c) maxillary sinus membrane elevation using a rotary-grind bur; (d) implant fixture placement; (e) suture with 4-0 Ethilon; (f) suture removal after 1 week; (g) the second surgery; and (h) final prosthesis loading.

**Figure 3:**
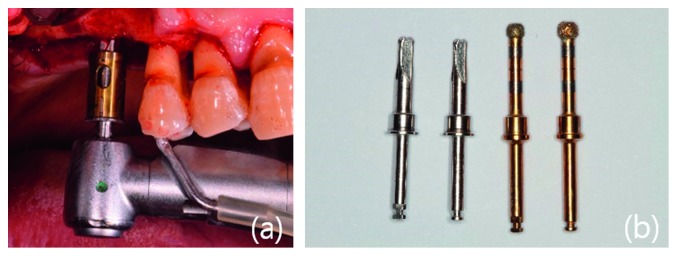
(a) Osteotomy preparation in the sinus floor using a rotary-grind bur. (b) The two drills on the left are CAS drills (reamer) and the two drills on the right are DASK drills (sinus bur).

The surgical site was sterilized, and infiltration anesthesia was administered using 2% lidocaine hydrochloride with epinephrine (1 : 1,00,000; Yuhan, Korea). After a crestal incision was made, full-thickness elevation was performed. The Crestal Approach Sinus Kit (CAS, Osstem Implant, Korea) and Dentium Advanced Sinus Kit (DASK, Dentium, Korea) were used, according to the manufacturer's instructions and the previous study [17]. Briefly, after using a pilot drill, a 2.0 mm twist drill was used to drill 1-2 mm shorter than the remaining alveolar bone height. Ø2.8 and Ø3.1 CAS drills with stopper were sequentially used to completely grind the cortical bone. Stopper systems with 1 mm increments were particularly useful when the bone height was not sufficient. If decortication of the sinus floor could not be achieved readily using the CAS drill alone, an additional drill from the DASK was used. The speed of the drill was maintained at 400∼600 rpm during the process. The sensation of a slight drop suddenly occurred when the sinus floor was completely grinded. Round shape of the drill top of CAS drill or diamond-coated drill in DASK can minimize the possibility of puncturing the sinus membranes. Sinus membrane perforation was checked using the Valsalva maneuver. The depth gauge with round tip in the kit was placed on the margin of the osteotomy, and the sinus membrane was carefully gently detached. To fully elevate membranes to the desired height, the bone graft was filled with bone carrier, and the bone graft was pushed into the sinus with bone condenser with stopper. A bone spreader was then used to laterally spread the bone graft material. Repeating this process, the membrane was sufficiently elevated by the bone graft material (1-2 mm longer than the implant length). We used MBCP (biphasic calcium phosphate, Biomatlante, France), OCS-B (deproteinized bovine bone, NIBEC, Korea), and ICB (allogenic cancellous bone, Rocky Mountain Tissue Bank, Aurora, CO, USA) alone or in combination (Table 2). SLA-surface implants (TS III, US II, Osstem Implant, Korea), with diameters of 4.0-5.0 mm and lengths of 8.5–10.0 mm, were used. Depending on the primary stability, implant placement was performed via a 1- or 2-stage technique. Suturing was performed using nonabsorbable sutures (4-0 Ethilon; Ethicon, OH, USA) (Figures 2 and 3).

A healing period of 6 months was permitted in the 1-stage technique; in contrast, a second surgery was performed after 4–6 months of healing in the 2-stage technique. The implant stability quotient (ISQ) was measured twice: once after implant placement (ISQ 1) and once immediately before impression taking of the final prosthesis (ISQ 2).

### 2.3. Evaluation of Marginal Bone Resorption after Final Prosthesis Loading

After loading the final prosthesis, the patients were instructed to make regular visits to the Department of Periodontology and Prosthodontics at 3- to 6-month intervals. During these visits, marginal bone resorption at the mesial and distal aspects of the implant was measured from parallel periapical radiographs; mean bone resorption values were recorded.

## 3. Results

A total of 10 implants were placed in 10 patients (Table 2). The mean residual bone height before implant placement was 3.41 ± 0.53 mm (range: 2.37–3.82 mm). Perforation of the sinus membrane did not occur during the procedures, and the patients experienced no severe pain, swelling, or discomfort after the procedure. Of 10 implants, eight were placed via the 2-stage technique, while two were placed via the 1-stage technique. From implant placement to final prosthesis loading, a mean healing period of 5.0 ± 0.8 months was observed (range: 4–6 months). The mean ISQ 1 was 63.4 ± 12.1, while the mean ISQ 2 increased to 77.6 ± 5.8 (Table 2).

**Figure 4:**
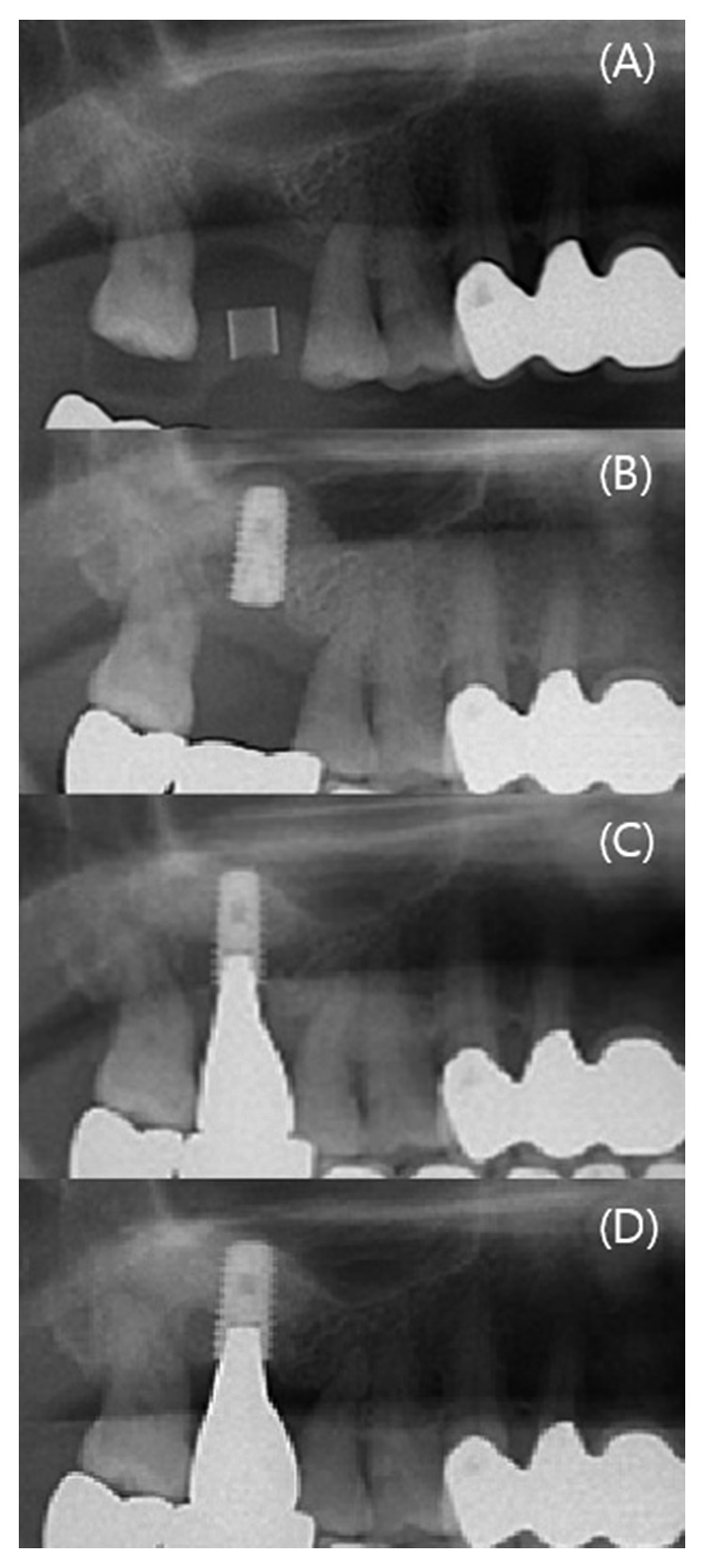
Partial view of panorama, according to procedure period. (A) Preoperative image; (B) maxillary sinus membrane elevation and implant placement; (C) final prosthesis loading; and (D) 6 months after the final prosthesis loading.

The mean follow-up observation period after final prosthesis loading was 12.0 ± 9.4 months (range: 4–34 months); during this period, no gingival inflammation, radiolucency, or implant mobility was observed. The mean marginal bone resorption was 0.23 ± 0.18 mm (range: 0.00–0.48 mm), as measured on periapical radiographs (Table 3, Figure 4).

## 4. Discussion

In the present study, successful outcomes were achieved via the alveolar crestal approach, using an RGB for MSME in patients who exhibited residual bone height of <4 mm. Although the number of patients was small and patients with systemic diseases were included in the study, our results demonstrated that implant treatment can be successfully performed using the alveolar crestal approach, even at lower alveolar bone heights.

For ISQ values measured immediately after implant placement, Patel et al. [21] reported a mean ISQ of 68.9 ± 1.6 for the lateral window approach, with a residual bone height of 3.0–7.9 mm. Additionally, Lai et al. [22] reported a mean ISQ of 68.0 using the osteotome technique, with a residual bone height of 4–8 mm. In the present study, the mean ISQ 1 was 63.4 ± 12.1, which was slightly lower than the mean ISQ reported in previous studies; however, the final value (77.6 ± 5.8 (ISQ 2)) was stable and demonstrated an appropriate value for osseointegration. In addition, in a patient with residual bone height of 2.37 mm and type 4 bone quality, the ISQ markedly increased from 42 to 74. As all patients exhibited a residual bone height of <4 mm, satisfactory stability may thus be achieved using an RGB; we suspect that even when primary stability is poor because of low alveolar bone height, sufficient osseointegration can still be achieved through the use of an RGB. Because the procedure was performed with a low alveolar bone height, most cases were performed by 2-stage technique, and 1-stage technique was rarely performed. Three cases (patient number 8, 9, and 10) that did not measure ISQ 1 were included in this study because they showed successful results of ISQ 2 even though we did not know a clear initial value. This study only explained that there were two different procedures involved in the clinic. It was not intended to claim the difference between the two procedures (1-stage or 2-stage).

Marginal bone resorption is a characteristic complication of implant treatment. Importantly, the degree of marginal bone resorption varies with differences in residual bone height. According to a study by Gonzalez et al. [20], who used the alveolar crestal approach by microsurgery, marginal bone resorption was 0.55 mm at a residual bone height of ≤4 mm and 0.07 mm at a residual bone height of ≥4 mm over an average of 29.7 months after surgery (range: 6–100 months). In the present study, the marginal bone resorption after final prosthesis loading was 0.23 ± 0.18 mm, which was less than the resorption reported in prior studies; this may be a consequence of the relatively short duration of this investigation. However, in three of 10 implants that were followed up after >1 year (13–34 months), marginal bone resorption did not exceed 1.5 mm, and the remaining seven implants exhibited marginal bone resorption of ≤0.5 mm.

There have been many studies investigating MSME via the alveolar crestal approach, using an osteotome on the maxillary posterior area with low residual bone availability; however, these investigations have reported conflicting results. Gonzalez et al. [20] reported that the implant success rates of MSME via the osteotome technique were 100% and 98.51% when the residual bone height was <4 mm and ≥4 mm, respectively. In that study, the most important factor in successful implantation was the achievement of proper stability in a low residual bone; notably, primary stability can be obtained even in a thin alveolar bone because it is provided by the ubiquitous presence of cortical bone at the crestal aspect of the ridge. However, other studies have insisted that residual bone height has a significant impact on the outcome of MSME. Rosen et al. [15] reported that the implant survival rate for a residual bone height of ≥5 mm was 96%; however, the rate decreased to 85.7% when the height was ≤4 mm. In addition, Toffler [5] reported that the implant survival rate was 94.5% for a residual bone height of ≥5 mm, which decreased to 73.3% for a height of ≤4 mm. These conflicting results may arise from differences in the implant surfaces used in the study. The studies by Rosen et al. [15] and Toffler [5] included implants that were mainly used in the past, such as machined surface or titanium plasma-sprayed implants. However, Gonzalez et al. [20] used sandblasted and acid-etched implants, which were developed relatively recently for research. The difference in surface treatment of these implants affects the initial fixation and osseointegration of the implant, even in areas where the residual bone height is insufficient, which may result in a difference in implant success rate.

Furthermore, there have been studies focused on avoiding the risks associated with the use of a mallet for MSME. Ahn et al. [17] used a reamer instead of a mallet, and reported a significant difference in the implant survival rate between residual bone height of <4 mm and ≥4 mm (92.7% and 96.4%, resp.), which is similar to the results of previous studies that involved a mallet. However, the implant survival rate increased to 96.2% when residual bone height was <4 mm and implants were placed with a length of 8–10 mm. This is likely a result of the difficulty in achieving elevation of the membrane by >10 mm using the crestal approach because of the resistance capacity of the Schneiderian membrane. Additionally, sinus membrane perforation occurred in only two of 98 (2.04%) patients with residual bone height of <4 mm. In another study, comparing osteotome and reamer technique using the crestal approach, 6.7% (three of 45) of patients experienced membrane perforation in the osteotome group, whereas 0.0% (zero of 40) of patients experienced membrane perforation in the reamer group [6]. In the present study, no sinus membrane perforation was observed in all patients who underwent a maxillary sinus elevation with residual alveolar bone height of <4 mm. Thus, using an RGB for MSME, the implant success rate was as high as the existing technique, and perforation was not observed despite insufficient residual bone height.

The amount of MSME through the crestal approach using an osteotome is 2.5–5.7 mm, whereas the amount that can be achieved using the lateral window approach is 10–12 mm [3–6]. Generally, the lateral window approach is recommended in cases where the residual bone height is low; however, in this study, we were able to perform implant placement via the crestal approach with an RGB on a residual bone height of <4 mm. Although the exact amount of elevation was not measured by CT in this study, panoramic radiographs revealed that the bone that was grafted to the apical area of the implant was well maintained throughout the study period. In this study, we used various bone graft materials including synthetic bone, allogenic bone, and heterogeneous bone except autogenous bone, but did not make a meaningful analysis in the results. The reason for this was because this study only aimed to demonstrate the viability of the crestal approach in MSME even with insufficient alveolar bone height. We are going to do further research on various variables.

## 5. Conclusions

Although there were limitations such as small sample size, short follow-up period, and insufficient consideration of various factors that can affect the success rate (anatomical shape of the sinus, type of bone graft materials, etc.), this study showed the possibility of MSME using an RGB on the maxillary posterior area that exhibited a residual bone height of <4 mm

## Figures and Tables

**Table 1:** Patient characteristics.

Patient	Age (years)	Sex	Medical history	Smoking
1	63	Male	Myocardial infarction (15 years ago)	No
2	71	Female	Hypertension	No
3	54	Female	Unremarkable	3-4 per day
4	67	Female	Hypertension	No
5	38	Male	Unremarkable	No
6	51	Male	Hypertension	No
7	42	Female	Unremarkable	3-4 per day
8	47	Female	Unremarkable	No
9	55	Female	Hyperlipidemia	No
10	54	Female	Hyperlipidemia	No

**Table 2:** Surgical attributes for each patient.

Patient	Surgical site	Residual bone height (mm)	Graft material	ISQ 1	ISQ 2	Method
1	#26	3.09	MBCP	75	83	2-stage
2	#16	3.82	OCS-B	68	83	2-stage
3	#16	3.56	OCS-B	52	73	2-stage
4	#17	2.62	MBCP	65	68	2-stage
5	#16	3.72	OCS-B	68	77	2-stage
6	#16	3.78	OCS-B	74	84	2-stage
7	#16	2.37	ICB + MBCP	42	74	2-stage
8	#27	3.70	MBCP	N/A	72	1-stage
9	#16	3.77	ICB + OCS-B	N/A	84	2-stage
10	#17	3.67	OCS-B	N/A	78	1-stage

ISQ 1: implant stability quotient measured during implant placement; ISQ 2: implant stability quotient measured immediately before impression taking for the final prosthesis; N/A: not applicable.

**Table 3:** Marginal bone resorption and follow-up observation period following final prosthesis loading.

Patient	Marginal bone resorption (mm)^*∗*^	Observation period (months)^*∗∗*^
1	0.00	4
2	0.12	6
3	0.00	8
4	0.36	11
5	0.22	7
6	0.28	13
7	0.00	4
8	0.48	34
9	0.38	22
10	0.43	11
Mean	0.23	12

^*∗*^Bone resorption as measured on periapical radiographs; ^*∗∗*^observation period following final prosthesis loading.

## Data Availability

The data are not available for public access because of patient privacy concerns.
